# A Case

**Published:** 1872-10

**Authors:** H. Scott


### A Case.

BY H SCOTT.

A few months since my attention was called to an infant of four or five weeks old, that it was said had cut a tooth. I found on the right side below, above the gum, a hard, white, lustrous point of the size of a grain of wheat, and appearing in every way like the cuspid of a molar well enameled. It was firmly fixed, not moving in the least when touched. The child is now probably five months old; the point has disappeared, and there is no dentition.

